# Editorial: New environmental pollutants, aging, and age-related diseases

**DOI:** 10.3389/fpubh.2026.1800119

**Published:** 2026-03-31

**Authors:** Xuefeng Lai, Wending Li, Yi Wang, Ling Zhang

**Affiliations:** 1 Department of Environmental Hygiene and Occupational Medicine, The Healthy Hubei Development and Social Progress Research Center of the Key Research Base of Humanities and Social Sciences in Hubei Province, Hubei Province Key Laboratory of Occupational Hazard Identification and Control, School of Public Health, Wuhan University of Science and Technology, Wuhan, China; 2 Department of Environmental Health Sciences, Mailman School of Public Health, Columbia University, New York, NY, United States; 3 Department of Epidemiology and Population Health, Albert Einstein College of Medicine, New York, NY, United States

**Keywords:** aging, age-related diseases, ethylene oxide (EO), microplastics, National Health and Nutrition Examination Survey (NHANES), new environmental pollutants, per- and polyfluoroalkyl substances (PFAS), phthalate

With the acceleration of global aging, age-related diseases have emerged as a growing public health challenge. Notably, genetic factors are estimated to contribute only about 20% to the aging process, while a substantial proportion of 80% can be attributed to environmental determinants, including social support systems, lifestyle factors, and physical environmental pollutants. Environmental exposures—from persistent organic pollutants and endocrine-disrupting chemicals to microplastics and emerging industrial compounds—represent an evolving and pervasive threat, whose impact on aging trajectories and geriatric diseases demands urgent investigation. However, their specific effects on aging biomarkers and related diseases remain poorly understood. This Research Topic aims to gather current research to elucidate how these pollutants influence biomarkers of aging and thereby exacerbate the risk of age-related diseases, providing novel evidence and mechanistic insights to support regulatory guidance and effective public health interventions for healthy aging and delayed onset of age-related diseases.

To that end, this Research Topic included a collection of eight studies that have approached this complex issue from multiple perspectives. A cross-sectional study conducted in Türkiye by Aslan and Kaplan sounded an alarm, revealing critically low awareness of the sources of exposure and health risks posed by common endocrine disruptors like bisphenols even among healthcare settings. This highlights an urgent need to enhance environmental health literacy by performing comprehensive educational interventions and policy reforms among both specialists and the general public (*Assessment of bisphenol-related knowledge and awareness among healthcare professionals: a cross-sectional analysis from Türkiye*: Aslan and Kaplan). Subsequent studies employed advanced biostatistical and machine learning techniques to delve deeper into specific pollutant-disease associations. For instance, research that utilized interpretable machine learning models on 4,450 participants from the National Health and Nutrition Examination Survey (NHANES, 2013–2018) uncovered complex and divergent relationships between different per- and polyfluoroalkyl substances (PFAS) and the risk of Chronic Obstructive Pulmonary Disease (COPD), successfully developing an online risk prediction tool for potential clinical use (*Developing an interpretable machine learning predictive model of chronic obstructive pulmonary disease by serum PFAS concentration*: Shao et al.). Another study, based on 2,519 participants from NHANES, 2013–2018, provided the first epidemiological evidence linking phthalate exposure to Circadian Syndrome (CircS), underscoring the combined health effects of mixture exposure (*Associations of urinary phthalate metabolites with Circadian Syndrome: evidence from NHANES*: Yi et al.).

Furthermore, other studies explored intermediate pathways and broader mechanisms through which emerging pollutants may accelerate aging-related outcomes. Zhang S. et al. utilized the NHANES database to investigate 30 epigenetic biomarkers as potential mediators linking environmental exposure to the risk of diabetes and cancer, analyzing the contributions of various epigenetic biomarkers to disease risk, and demonstrating the value of machine learning in deciphering complex biomarker networks (*The relationship between epigenetic biomarkers and the risk of diabetes and cancer: a machine learning modeling approach*: Zhang S. et al.). Kong and Jin applied Weighted Quantile Sum (WQS) and Bayesian Kernel Machine Regression (BKMR) to directly assess the association of common environmental chemicals (perchlorate, nitrate, and thiocyanate) with “phenotypic age” and “biological age,” offering a novel perspective on how chemical exposure may influence an individual's rate of aging (*Environmental exposure to perchlorate, nitrate, and thiocyanate in relation to biological aging in U.S. adults, a cross-sectional NHANES study*: Kong and Jin).

Regarding disease-specific research, a study finds a non-linear (J-shaped) association between ethylene oxide (EO), a widely used disinfectant, and the risk of Osteoarthritis (OA), which is more pronounced in individuals with specific nutritional deficiencies or in younger age groups (*Association between ethylene oxide exposure and osteoarthritis risk: an analysis of NHANES data (2013–2014 and 2017–2018*: Li Z. et al.). Li D. et al. explored a subtle non-linear relationship between the heavy metal cadmium and non-melanoma skin cancer (NMSC), suggesting potential risks even at low-level exposure (*Is there an association of blood cadmium level with nonmelanoma skin cancer: results from a cross-sectional study*). Finally, as a comprehensive review, Zhang X. et al. systematically outlines the pathways of microplastics into the human body, their mechanisms for inducing toxicity, and their synergistic effects with other pollutants, charting a course for future research in this emerging field (*Microplastics and human health: unraveling the toxicological pathways and implications for public health*: Zhang X. et al.).

Collectively, studies in this Research Topic demonstrated that emerging environmental pollutants are key factors driving the aging process and age-related diseases. These works not only established new associations between specific chemicals and health outcomes but also deepen our understanding of the complexity of exposure-response relationships through innovative methodologies, such as explainable machine learning and mixture analysis models. However, a gap remains between knowledge and action. To bridge this gap, whether by addressing knowledge deficits among healthcare workers or by updating public health policies and regulatory standards based on new evidence, it requires urgent, interdisciplinary efforts. We hope this Research Topic stimulates broader and deeper discussion and research, ultimately contributing to the building of a societal environment that reduces harmful exposures, promotes healthy aging, and ultimately delays the onset of age-related diseases.

